# Cassaine Diterpenoid Amide from Stem Bark of *Erythrophleum fordii* Suppresses Cytotoxic and Induces Apoptosis of Human Leukemia Cells

**DOI:** 10.3390/molecules25143304

**Published:** 2020-07-21

**Authors:** Tu Thanh Thi Nguyen, Dao Cuong To, Phuong Hien Thi Vo, Thanh Hoa Tran, Phi Hung Nguyen, Hien Minh Nguyen, Manh Hung Tran

**Affiliations:** 1 Traditional Medicine Faculty, Hanoi Medical University, 1 Ton That Tung street, Dong Da District, Hanoi 116001, Vietnam; thanhtu@hmu.edu.vn; 2 Faculty of Pharmacy, Phenikaa University, Yen Nghia, Ha Dong district, Hanoi 12116, Vietnam; cuong.todao@phenikaa-uni.edu.vn; 3 Phenikaa Research and Technology Institute (PRATI), A&A Green Phoenix Group JSC, 167 Hoang Ngan, Cau Giay District, Hanoi 11313, Vietnam; 4 Faculty of Biology and Biotechnology, University of Science, Vietnam National University Hochiminh City, 227 Nguyen Van Cu street, District 5, Ho Chi Minh City 748000, Vietnam; phuonghienvt1992@gmail.com; 5 Biomedical Science Department, VNUK Institute for Research & Executive Education, The University of Danang, 158A Le Loi street, Hai Chau District, Danang City 551000, Vietnam; hoa.tran@vnuk.edu.vn; 6 Institute of Natural Products Chemistry, Vietnam Academy of Science and Technology (VAST), 18 Hoang Quoc Viet street, Cau Giay District, Hanoi 122100, Vietnam; nguyenphihung1002@gmail.com; 7 Faculty of Pharmacy, Ton Duc Thang University, 19 Nguyen Huu Tho street, District 7, Ho Chi Minh City 758307, Vietnam; nguyenminhhien@tdtu.edu.vn; 8 Faculty of Chemistry, University of Science, Vietnam National University Ho Chi Minh city, 227 Nguyen Van Cu Street, District 5, Ho Chi Minh City 748000, Vietnam

**Keywords:** *Erythrophleum fordii* oliver, caesalpinioideae, cassaine diterpenoid, 3β-acetyl-nor-erythrophlamide, human leukemia cancer cells, molecular docking

## Abstract

Cassaine diterpenoids amides from the stem bark of Vietnamese *Erythrophleum fordii* Oliver were screened for their cytotoxic activity against human cancer cells. The cell proliferation assay results showed that, among the active compounds, 3β-acetyl-nor-erythrophlamide (3AEP) exhibited the most potential cytotoxicity against human leukemia HL-60 and KG cells with IC_50_ values of 12.0 ± 1.2 and 18.1 ± 2.7 µM, respectively. Treatment of 3AEP resulted in the apoptosis of HL-60 cells via the activation of caspase 3, and poly (ADP-ribose) polymerase (PARP). Molecular docking in silico results showed that the 3AEP can bind to both the procaspase-3 allosteric site and the PARP-1 active site, with binding energies of −7.51 and −9.63 kcal/mol respectively. These results indicated that the stem bark of Vietnamese *E. fordii* and its cassaine diterpenoid amides may be useful in the apoptosis induction of human leukemia cancer cells.

## 1. Introduction

*Erythrophleum fordii* Oliver is a large tree belonging to the Leguminosae. It is a wood plant that can reach over 30 m in height, primarily distributed in Vietnam, Taiwan, and China. It contains many acrid substances used for dyeing [1]. In Vietnam, the wood of *E. fordii* is one of four types of famous Vietnamese timbers using in housing construction from the ancient times (Dinh, Lim, Sen, and Tau) [1]. *E. fordii* is using as a herbal ingredient in traditional Chinese medicine to promote blood circulation [2]. *E. fordii* contains cassaine diterpenoid amide alkaloids, triterpenoids and steroids. Many of them show pharmaceutical and biological activity as anticancer, antioxidant, and anti-inflammatory agents [2,3,4,5,6]. Previously, we had isolated many mono- and dimeric cassaine diterpenoid amides from the stem bark of Vietnamese *E. fordii*. Of these, monocassaine diterpenoid amides showed a potential impact on Matrigel endothelial formation and capillary structure formation in human umbilical vascular endothelial cells [7]. Meanwhile, the diterpenoid- diterpenoid dimer amides exhibited a strong cytotoxic impact on PC-3 prostate cancer cells through an apoptosis pathway [8]. In addition, we reported several new cassaine diterpenoids and cassaine diterpenoid amines isolated from Vietnamese *E. fordii*. These compounds were tested against non-small cell lung cancer cell lines such as A549, NCI-H1975, and NCI-H1229. The results showed that cassaine diterpene amines exhibited potential cytotoxic activity against these cancer lines with IC_50_ values between 0.4 and 5.9μM. Interestingly, erythroformine B significantly induced apoptosis in A549, NCI-H1975, and NCI-H1229 cells [9]. From the seeds of *E. fordii* collected in China, several cassaine diterpenoids were isolated and tested for cytotoxicity against MCF-7 and A549 cancer cells [10]. Recently, Li et al., reported that the cassane diterpenoids displayed potential antiviral activities opposing influenza and coxsackie virus [6]. In our preliminary screening, a serial of cassaine diterpenoid amides isolated from Vietnamese *E. fordii* showed cytotoxic activity against human cancer cells. In this study, we examined the cytotoxic activity of an active cassaine diterpenoid amide named 3β-acetyl-nor-erythrophlamide (3AEP) against human leukemia cancer cells.

## 2. Results and Discussion

**Figure 1:**
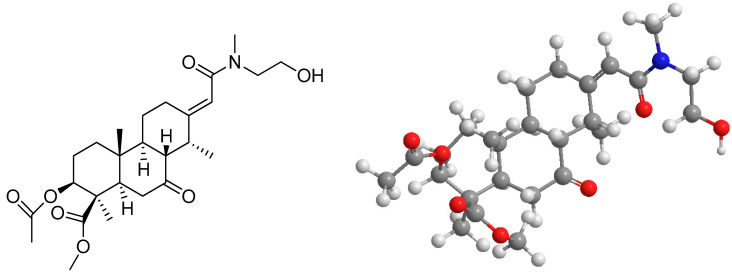
1D and 2D chemical structure of 3AEP.

The compound 3β-acetyl-nor-erythrophlamide (3AEP, Figure 1) and the other cassaine diterpenoid amides were isolated from a methanol extract of the stem bark of Vietnamese *E. fordii* by our research group. The compounds were identified by their physicochemical and spectroscopic data (IR, UV, MS, 1D and 2D NMR). Chemically, among them, six are secondary metabolites belonging to the monocassaine-type diterpenoid amide type such as erythroformide, nor-erythrophlamide, 3β-acetyl-nor-erythrophlamide (Figure 1), 6α-hydroxy-nor-cassamide, 3β-acetoxy-norcassamide, and nor-cassamide [7]. In addition, the other two compounds were identified as erythrophlesin H and erythrophlesin I which structures are unique due to the signals observed in pairs in the NMR spectra [8]. Both of them had a dimeric structure consisting of two tricyclic diterpenoids that were constructed from two cassaine diterpenoids connected through an ester linkage in the presence of a 7-ketone group for both monomer left and right units [8].

To determine the cytotoxic activity of compounds, we performed a cell proliferation assay using a cell counting kit. The results showed that, among the active compounds, 3β-acetyl-nor-erythrophlamide (3AEP) exhibited moderate cytotoxicity against leukemia HL-60 cancer cells with an IC_50_ value of 12.0 ± 1.2 µM. This mono amide also showed cytotoxicity against KG1 and Hela cells with IC_50_ values of 18.1 ± 2.7 and 16.5 ± 2.7 µM, respectively. Among the other compounds, the dimer amide erythrophlesin H exhibited significant cytotoxic activity against OVCAR8 cells with an IC_50_ value of 16.7 ± 1.3 µM. The other compounds showed weak or no cytotoxic activity. 3AEP also showed no cytotoxic to normal human embryonic kidney cells (HEk293) (Table 1).

Caspase 3 is a member of the cysteine-aspartic acid protease family. Caspases exist as inactive proenzyme forms that encounter proteolytic processing at conserved aspartic residues to manifest large and small subunits. These subunits dimerize to the active enzyme form. Usually, sequential activation of caspases played a central role in the execution-phase of cell apoptosis. For example, activated caspase 3 led to the activation of the other caspases 6 and 7, these processes cleave multiple structural and regulatory proteins, and are critical for cell survival and maintenance [11]. Caspase 3 was identified as the most important executioner enzyme, activated by both intrinsic and extrinsic pathways [12].

**Figure 2:**
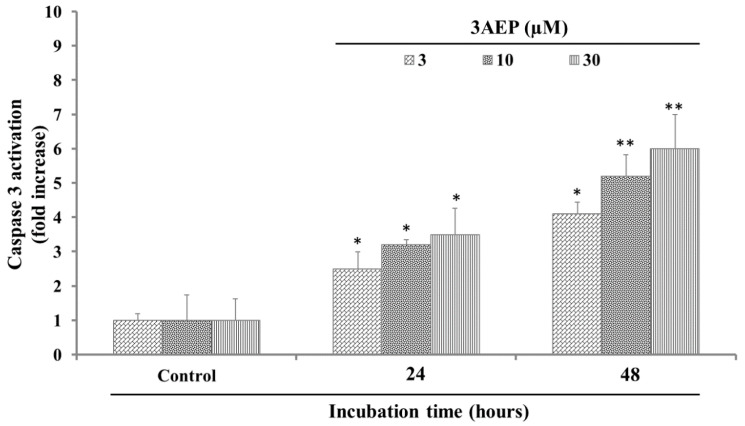
Effect of 3AEP on caspase-3 activation in HL-60 cells. Cells were incubated with 3AEP (0–30 μM) for 24 and 48 h. The cell lysates were incubated at 37 °C with caspase-3 substrate (Ac-DEVD-AFC) for 1 h. The fluorescence intensity of the cell lysates was measured to determine the caspase-3 activity. The blank group was used as 0.1% DMSO-treated cells. Data are presented as the mean ± SD of results from three independent experiments (* *p <* 0.01; ** *p <* 0.05).

When caspase 3 was in activation mode, it caused cell death by an apoptosis pathway via cleaving proteins into heterozygous substances [12]. In our experiment, since 3AEP showed the most potential cytotoxicity against HL-60, it was consequently selected for the next experiments. 3AEP (0–30 µM) was added to HL-60 cells (1 × 10^6^ well) followed by incubation for 24, and 48 h. After that, the caspase 3 activation in HL-60 cells was measured by the levels of Ac-Asp-Glu-Val-Asp- 8-amino-4-trifluoromethylcoumarin (Av-DEVD-AFC). The results in Figure 2 show that 3AEP activated caspase 3 activity by increasing the cleaved caspase 3 level from 2 to 4 folds in 24 h incubation. In addition, after 48 h, this compound activated caspase 3 into a cleaved form with 4 to 7-fold higher activity compare to the control (Figure 2). These results indicated that 3AEP could increase the activation of caspase 3 in a dose and time dependent manner.

**Figure 3:**
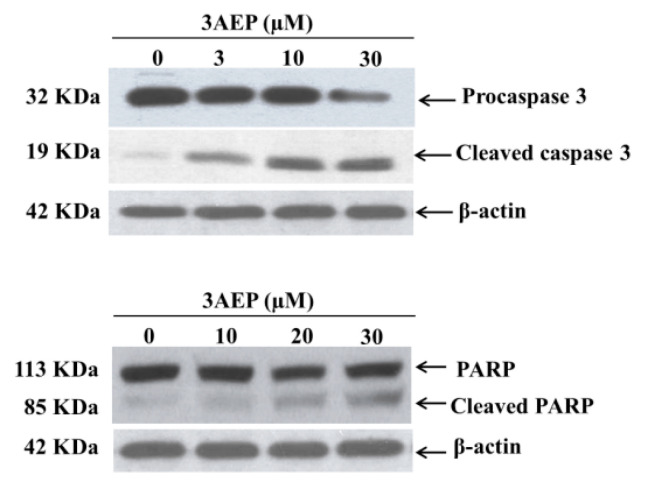
Caspase 3 and PARP degradation protein expression by 3AEP in HL-60. Cells were treated with 3AEP (0–30 µM) for 48 h. Protein 50 µg/lane from cells lysates were electrophoresed on SDS-PAGE gels, and then transferred to total blot PVDF membranes. β-actin was used as a control, (–), 0.1% DMSO-treated cells. The experiments were carried out in three experiments.

To identify whether 3AEP could induce HL-60 cell death by an apoptotic pathway, we measured the protein levels of caspase 3 and cleaved poly(ADP-ribose) polymerase (PARP) using western blotting analysis. PARP is a protein family that plays an important role in many cellular processes, such as DNA repair, genomic stability, and programmed cell death. Catalyzing caspase-3 generally leads to the cleavage of PARP, and this process which plays a positive regulatory role in the apoptosis of several cells, is considered as a biomarker for the detection of apoptosis [13]. The HL-60 cells were treated with 3AEP (0–30 µM) for 48 h. Protein (50 µg/lane) from cells lysates was electrophoresed on SDS-PAGE gels, and then transferred to total blot PVDF membranes. As shown in Figure 3, 3AEP induced the caspase 3 transform into its cleaved form in a dose-dependent manner. Exposure of 3AEP for 48 h also triggered PARP into cleavage form (Figure 3).

**Figure 4:**
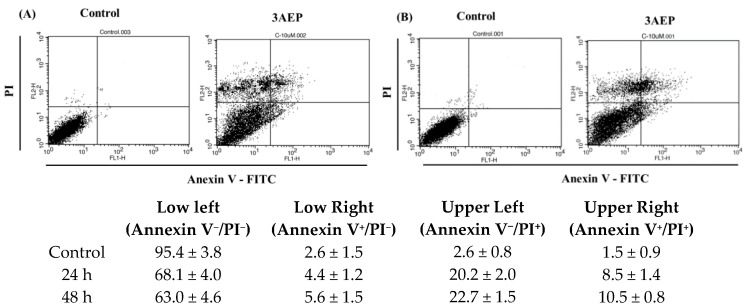
Flow cytometry analysis of 3AEP on HL-60 cells in 24 h (**A**) and 48 h (**B**). The results were expressed as the mean ± standard deviation (SD) of three replicates.

In the next study, flow cytometry analysis was performed using an Annexin V/PI double staining assay for 24 h (A) and 48 h (B) in the presence of 3AEP (10 μM). HL-60 cells were harvested and stained with PI and Annexin V-FITC in darkness for 15 min, and subjected to flow cytometry using a BD Biosciences platform. Early and late apoptotic and necrotic cells were identified clearly (Figure 4). Treatment of HL-60 cells with 3AEP (10 μM) has increased the population of Annexin-V^+^/PI^+^ (late apoptosis) and Annexin-V^+^/PI^−^ (early apoptosis) cells in a time-dependent manner. Interestingly, the population of Annexin-V^−^/PI^+^ (necrosis) has been shown with about 20.2 to 22.7%. Together with the western blotting results that both the cleavage of capase 3 and PARP, the two important apoptotic marker proteins, it is worthy to note that 3AEP could induce apoptosis in HL-60 cells via caspase pathways.

**Figure 5:**
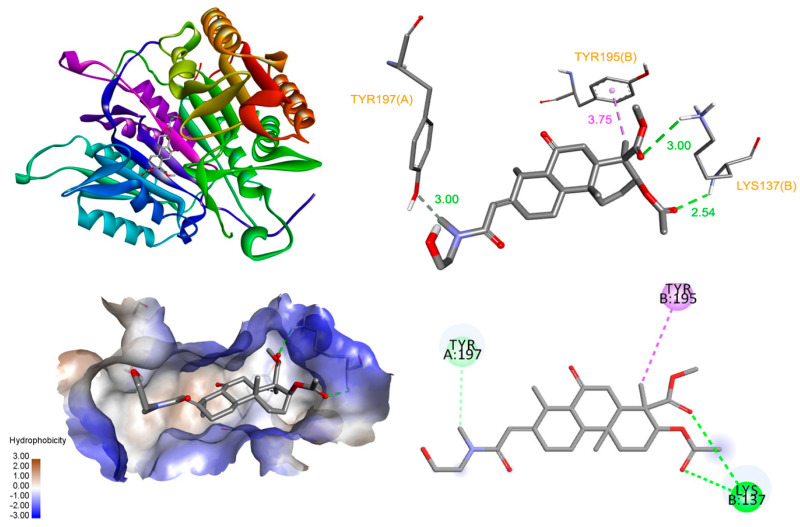
Interaction diagram of 3AEP with caspase-3.

Based on these in vitro studies, we investigated the binding of 3AEP with caspase-3 and PARP-1 proteins using molecular docking simulation [14]. The allosteric site of procaspase-3, at the dimer interface of caspase-3, has been suggested to play a critical role in the active site formation of caspase-3, and the efficient binding of an activator at this site can stabilize the active caspase-3 conformer [15,16]. The docked pose of 3AEP showed that it can bind to the interface with a binding energy of −7.51 kcal/mol, forming hydrogen bonds with Tyr197 (3.00 Å), and Lys137 (2.54 Å). However, only one hydrophobic contacting at Tyr195 appeared between the compound and the interface of enzyme (Figure 5).

**Figure 6:**
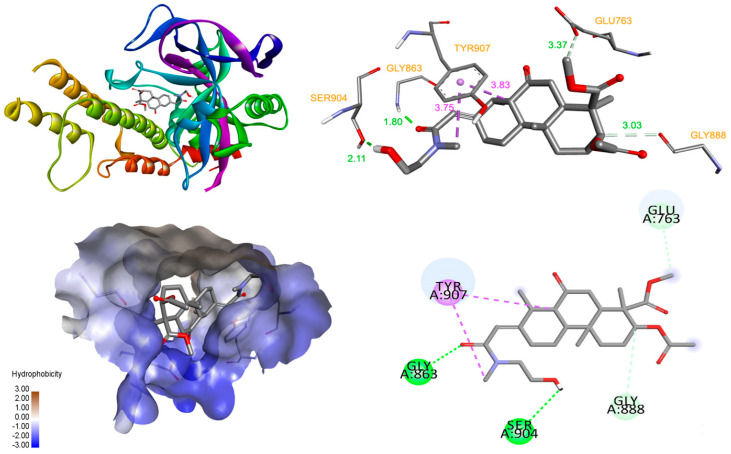
Interaction diagram of compound with PARP-1.

When the 3AEP was docked into the catalytic active site of PARP-1, the results indicated a high affinity, with a binding energy of −9.63 kcal/mol. The detailed interaction diagram (Figure 5) showed that 3AEP has several interactions with PARP-1. The hydroxyl, methoxy, and carbonyl groups of the 3AEP were found to form hydrogen bonds with Gly863 (1.8 Å), Ser904 (2.11 Å), Glu763 (3.37 Å), and Gly888 (3.03 Å) at both monomers. In the nicotinamide-binding pocket of PARP-1, Tyr907 interacted with nicotinamide ring to maintain NAD+ in this pocket [17]. Therefore, the binding of the compound with the active site of PARP-1 occured with high affinity and many interactions. One of these interactions is the critical residue Tyr907 at hydrophobic, which might interfere with the binding of NAD+ and affect the activity of PARP-1 [18]. These docking results suggested that PARP-1 target is more appropriate than caspase-3. The results demonstrated that 3AEP can bind to both the procaspase-3 allosteric site and the PARP-1 active site (Figure 6). However, further studies such as detail PARP-1 inhibition and PAPR-1 binding assays should be performed to validate this finding.

Human leukemia is a malignant disorder of lymphoid progenitor cells that impacts both children and adults [19]. The prognosis of leukemia patients has improved over the past decades [20]. Among the treatments, chemotherapy is frequently used as a main therapy for leukemia [21]. Despite the significant progress, during the treatment of this type of cancer, drug resistance is a core problem. Due to the limitation of the acute and chronic toxic impacts associated with current treatment agents, the potential methods for dose escalation using current chemotherapeutics in order to overcome drug resistance are enormously challenging [22]. Therefore, new treatment modalities are required, especially those based on finding new of natural products with suitable activity [23,24].

It is well-known that natural products, including natural plants, marine and bacterial organisms, have been used for many therapeutic purposes. These products, including extracts and pure compounds, are considered less toxic than synthetic chemical agents, making them safe resources that could be used as a main mediators for the development of anticancer drugs. In our study, we have been identified that a cassaine diterpenoid amide, 3AEP, isolated from the bark of Vietnamese *E. fordii* exhibited cytotoxicity against HL-60 cancer cells and other cell lines. The cell proliferation assay showed that 3AEP is more active than other cassaine diterpenoid amides not only on HL-60 but also on the other cancer cells (Table 1). Previously, 3AEP was found to exert antitumor activity, whereby this alkaloid inhibited the vascular endothelial growth factor mediated proliferation, migration, invasion, and capillary-like tube formation of human umbilical vascular endothelial cells (HUVECs). 3AEP then blocked angiogenesis *in vivo*, and suppressed tumor angiogenesis and human lung adenocarcinoma growth in the xenograft tumor model *in vitro* [25]. Interestingly, 3AEP induced cell apoptosis related to the level of caspase-8 and Bcl-2 expression of human lung cancer cells and lymphoma cells [26]. This study reports for the first time that 3AEP induced apoptosis, displaying caspase 3 activation and PARP inhibition in human leukemia HL-60 cells. Chemically, this compound has a cassaine diterpenoid amide skeleton without a hydroxyl group at C-5, but it has linkage with 3β-acetyl and 4β-methoxycarbonyl groups in C3 and C4, respectively. Therefore, we speculate that these characteristics might be considered as the active groups of cassaine diterpenoid amide which could strengthen the cytotoxic activity. This information also supports semi-synthesis to optimize the cytotoxic activity of 3AEP and its derivatives. Taken together, in accordance with the previous results, the findings of our experiment provided provide evidence suggesting 3AEP is a potential therapeutic agent for the treatment of human leukemia.

## 3. Materials and Methods

### 3.1. Cell Lines and Culture

The cancer cells as ovarian (OVCAR 8), leukemia cancer cell lines HL-60 (human acute promyelocytic leukemia), KG1 (human acute myelogenous leukemia), pancreatic (PANC-1), pancreas epithelial cell (MIA PACA 2), cervical cancer (Hela) cells, and a normal human embryonic kidney cells (HEk293) were obtained from the American Type Culture Collection (ATCC, Manassas, VA, USA). The cells were maintained in DMEM (Gibco BRL, New York, NY, USA) with 10% fetal bovine serum (FBS) supplemented with 2% penicillin and 100 μg/mL of streptomycin at 37 °C in a 95% humidified atmosphere containing 5% CO_2_.

### 3.2. Cell Proliferation Activity Assay

Cell proliferation activity was determined against cancer cell lines using a cell counting kit (Dojindo Molecular Technologies, Inc., Rockville, MD, USA) with a slight modification. Detail proliferation assay was reported in supporting information file.

### 3.3. Preparation of Total Cell Extract and Immuno Blot Analysis

Immunoprecipitation and western blotting were described in our previous paper. Briefly, immunoblot analysis and immunoreactive proteins were visualized by an enhanced chemiluminescence (ECL) procedure according to the manufacturer’s protocol. HL-60 and KG cells (5 × 10^5^ cells/mL) were treated with active isolated compounds (3–30 μM) for 48 h at 37 °C. Cell lysates were prepared in 100 μL of lysis buffer (Sigma, Ronkonkoma, NY, USA) containing a protease inhibitor cocktail (Roche, Mannheim, Germany). The protein extract (50 μg/well) was separated by SDS-PAGE and then transferred onto PVDF membranes (Bio-Rad, Hercules, CA, USA). The detailed membranes blocking, primary antibodies, immunoreactive proteins visualization, anti-β-actin detection were reported in supporting information files.

### 3.4. Detection of Apoptosis by Double Stanning

An Annexin V-FITC/PI staining kit was used to detect the phosphatidylserine translocation, an important characteristic at an early stage of cell apoptosis. Briefly, HL-60 cancer cells were seeded in 6 well plates at a density of 2 × 10^5^ cells/mL and incubated for 24 h. After that, cells were treated with 3AEP for 24 and 48 h. The cells were collected and washed in PBS, then were resuspended in 195 μL binding buffer, and incubated with 10 μL Annexin V-FITC and 5 μL PI in the dark for 15 min. Thereafter, the solutions were immediately measured by FCM (Beckman, Fullerton, CA, USA).

### 3.5. Molecular Docking Simulation

The structure file of the compound was created using MarvinSketch (ChemAxon, Budapest, Hungary). The crystal structures of caspase-3 (PDB ID: 2J30) and PARP-1 (PDB ID: 5WS1) were obtained from Research Collaboratory for Structural Bioinformatics Protein Data Bank (RCSB PDB). Proteins were firstly edited using UCSF Chimera 1.14 by removing waters, rebuilding missing residues, and removing co-crystallized ligands, cofactors. After that, both protein and compound were processed using AutoDockTools 1.5.6. Ligand-binding sites and grid box parameters were defined based on potential binding cavities identified by MetaPocket2.0 server. Molecular docking studies were done by Autodock 4.2 using the Lamarckian genetic algorithm with default settings. The docking results were analyzed with the Discovery Studio Visualizer.

### 3.6. Statistical Analysis

Data are expressed as the mean ± standard deviation (SD). Graphs were plotted and statistical analyses were performed using SigmaPlot 6.0 and SigmaStat 3.1 (Systat Software, San Jose, CA, USA) and ANOVA followed by the Tukey’s test on the pre-validated data; or, alternatively using a Prism (GraphPad Software, San Diego, CA, USA) and an unpaired Student’s *t*-test (when comparing two groups) or one-way nonparametric ANOVA (Tukey’s test) followed by Bonferroni post hoc analysis (for the comparison of more than two groups) or correlation analysis. Statistical significance was set at *** *p* < 0.001; ** *p* < 0.05; * *p* < 0.1.

## 4. Conclusions

In conclusion, to the best of our knowledge, we exploded for the first time that a cassaine diterpenoid amide, 3AEP from the stem bark of Vietnamese *Erythrophleum fordii* Oliver, effectively suppressed HL-60 cancer cell growth by apoptosis. This compound not only significantly inhibited cell proliferation and activated caspase 3 but also upregulated the PARP protein levels in HL-60 cells dose-dependently. The docking simulation results indicated that 3AEP could bind to both the procaspase-3 allosteric site and the PARP-1 active site with binding energies of −7.51 and −9.63 kcal/mol respectively. These results suggested that the 3AEP might induce apoptosis in human leukemia cancer cell lines.

## Figures and Tables

**Table 1:** Cytotoxic activities of cassaine diterpenoid amides from *E. fordii* against human leukemia cancer cell lines.

Compounds	Cell Lines (IC_50_, µM) ^a^					
	OVCAR8	HL-60	KG1	PANC-1	MIA PACA2	Hela
Erythroformide	>30	23.2 ± 2.5	>30	>50	>50	>30
Nor-erythrophlamide	>30	25.7 ± 1.8	21.5 ± 1.3	>50	>50	20.5 ± 1.4
3β-Acetyl-nor-erythrophlamide	>30	12.0 ± 1.2 *	18.1 ± 2.7 **	>30	>30	16.5 ± 2.7
6α-Hydroxy-nor-cassamide	>30	25.7 ± 1.9	>50	>30	>30	>30
3β-Acetoxy-norcassamide	>50	28.5 ± 1.9	>30	>30	>30	>30
Nor-cassamide	>50	>50	>50	>50	>50	>50
Erythrophlesin H	16.7 ± 1.3	>50	28.5 ± 2.0	>30	>30	26.3 ± 4.0
Erythrophlesin I	21.5 ± 1.9	>50	>30	>30	>30	20.7 ± 1.8
Camptothecin ^b^	1.9 ± 0.1	1.7 ± 0.4	1.6 ± 0.5	6.5 ± 0.8	3.2 ± 0.7	0.8 ± 0.1

^a^ Results are expressed as IC_50_ in µM. The cytotoxicity effect was expressed as the mean ± standard deviation (SD) of three replicates. Statistical significance was accessed by Duncan’s multiple range tests (* *p* < 0.01; ** *p* < 0.05); ^b^ Positive control.
